# Editorial: New Therapeutic Approaches Against Inflammation and Immune Regulation in Metabolic Related Diseases

**DOI:** 10.3389/fphar.2022.878608

**Published:** 2022-03-10

**Authors:** Yao Lu, Liang Weng

**Affiliations:** ^1^ Clinical Research Center, The Third Xiangya Hospital, Central South University, Changsha, China; ^2^ Department of Oncology, Xiangya Cancer Center, Xiangya Hospital, Central South University, Changsha, China; ^3^ Key Laboratory of Molecular Radiation Oncology Hunan Province, Xiangya Hospital, Central South University, Changsha, China

**Keywords:** metabolic disease, inflamation, immune, cancer, diabetes, CVD (cardiovascular disease)

Abnormal metabolism accompanied by chronic low-grade inflammation plays a vital role in the cause and progression of many metabolic-related diseases. The crosstalk of metabolism and inflammation has attracted more and more attention in maintaining tissue and organ homeostasis in recent years. In this Research Topic of Frontiers in Pharmacology, several novel findings further extend our understanding on the role of metabolism and inflammation in diseases, with emphasis on diabetes, metabolic syndrome, cardiovascular disease, cancers and kidney disease progression and treatment.

Cardiovascular disease, especially atherosclerosis is highly related to dysregulated metabolism. In addition, several immune signaling pathways and immune cells are involved in the progression of atherosclerosis. In this Research Topic, Jiang et al., reported that Caspase-11-gasdermin D-mediated pyroptosis and the subsequent proinflammatory response in macrophages are involved in the pathogenesis of atherosclerosis. Liu et al. reported that a peptide named intermedin can prevent ox-LDL–induced macrophage inflammation by inhibiting FABP4, implicating the potential application of this peptide in atherosclerosis treatment in future. Furthermore, an excellent review by Leng et al. summarized the receptor interacting protein kinases 1/3 (RIPK1/3) mediated signaling pathways in cardiovascular diseases progression and treatment.

Diabetes and metabolic syndrome are prevalent worldwide and major public health problems, which affect the health of people and impair the quality of life. This Research Topic provided new insights into the interplay between metabolism and inflammation in these diseases. Four papers (Ding et al., Hu et al., Collado et al., and Dong et al.) reported the effect of atrial natriuretic peptide (ANP), unsaturated fat, the soluble dietary fiber polydextrose (PDX) and probiotic supplementation on diabetes and metabolic syndrome treatment with inhibition of inflammatory levels. In addition, a review by Geng et al. discussed the innate immune cellular molecular events in the local microenvironment of diabetic wounds and the potential of targeting these immune pathways and cell phenotypes in diabetic wound therapy.

Kidney removes metabolic wastes and reabsorbs water, minerals and nutrients to participate in whole-body homeostasis. Abnormal metabolism and immune system function can be found in many kidney diseases. In this Research Topic, two papers described the outcomes of surgical operation in kidney diseases. Guangyu et al. analyzed the cardiovascular and cerebrovascular risk of postoperative acute kidney injury (AKI) in surgical patients. Single-cell transcriptomics sequencing is a powerful method to identify the composition of cell types in samples. By using this method, Zhuang et al. analyzed the immune cell subpopulations in the end-stage renal disease patients who received kidney transplantation, and revealed a novel B-cell subset (CD19^+^IGLC3^low^IGKC^high^TCL1A^−^CD127^+^) in renal allograft recipients with immune accommodation. In addition, in the treatment of IgA nephropathy, Liu et al. revealed that Astragaloside IV could inhibit the secretion of galactose-deficient IgA1, which is the main cause for IgA nephropathy.

Cancer is long considered as a metabolic-related disease. As early as in 1920s, the Warburg effect, also known as aerobic glycolysis, was discovered to support cancer cell growth. In recent years, with the advances of technique in metabolomics, several other metabolic pathways were identified to be involved in tumorigenesis. Furthermore, dysregulated metabolism was also shown to modulate tumor immune surveillance. In this research topic, two papers reported a set of genes related to immune regulation and metabolism in predicting the prognosis of cancers. Liu et al. found the levels of T-cell exhaustion-associated genes and the abundance of immune cells were elevated under high HIF1A expression in glioma. Chen et al. established a prediction model with four metabolic genes as a reliable prognostic tool to accurately predict the prognosis of LUAD. Finally, a state-of-the-art review summarized the clinical efficacy and safety of anti-PD1 and anti-PD-L1 immunotherapy.

Taken together, the researches presented in this Research Topic provide the new insight into the role of metabolism and inflammation in metabolic diseases.

